# Knowledge, Attitudes, and Beliefs about Relapse Prevention Research Involving Bupropion among Current and Former Pregnant Individuals Who Smoke

**DOI:** 10.1155/2022/1925071

**Published:** 2022-12-16

**Authors:** Melissa Adkins-Hempel, Sandra J. Japuntich, Janet Thomas, Pearl Fang, Katherine Harrison, Rebecca L. Emery Tavernier, Jonathan P. Winickoff, Michael Kotlyar, Sharon Allen

**Affiliations:** ^1^Hennepin Healthcare Research Institute, USA; ^2^Hennepin Healthcare, USA; ^3^University of Minnesota Medical School, USA; ^4^University of Minnesota Medical School, Duluth Campus, USA; ^5^Massachusetts General Hospital, USA; ^6^Harvard Medical School, USA; ^7^University of Minnesota College of Pharmacy, USA

## Abstract

**Introduction:**

While many individuals quit smoking during pregnancy, most relapse within one year postpartum. Research into methods to decrease smoking relapse postpartum has been hampered by difficulties with recruitment.

**Method:**

We conducted individual interviews with pregnant women (*N* = 22) who were interested in quitting smoking while pregnant about their attitudes regarding smoking and quitting during pregnancy, clinical trial participation, and smoking cessation medication use.

**Results:**

Participants were aware of the risks of smoking while pregnant. Many wanted to quit smoking before delivery. Few used empirically supported treatments to quit. While research was viewed positively, interest in taking on new commitments postpartum and taking a medication to prevent relapse was low. Medication concerns were evident among most participants, especially among those planning to breastfeed. Further, several women noted medication was unnecessary, as they did not believe they would relapse postpartum. Financial incentives, childcare, and fewer and/or remote visits were identified as facilitators to participating in research. However, these factors did not outweigh women's concerns about medication use and time commitments.

**Conclusions:**

Women are aware that quitting smoking during pregnancy and remaining smoke-free postpartum are important. However, beliefs that personal relapse risk is low and that medications are dangerous reduced enthusiasm for taking medication for postpartum relapse prevention. Future medication trials should educate women about the high likelihood of relapse, prepare to answer detailed questions about risks of cessation medications, and connect with participants' clinicians. For new mothers, studies conducted remotely with few scheduled appointments would reduce barriers to participation.

## 1. Introduction

Cigarette smoking during pregnancy has adverse fetal and child outcomes, including spontaneous abortion, ectopic pregnancy, preterm delivery, and low birthweight [1–3]. Approximately 54% of women quit smoking prior to or during pregnancy [4]. Unfortunately, around 50% will relapse within the first 6 months postpartum, with the number climbing even higher at one year postpartum [5–9]. Smoking postpartum increases the risk of smoking-related disease for women and has negative effects on childhood development due to secondhand smoke (e.g., increased risk of sudden infant death syndrome, respiratory infections, and other diseases) [4, 10]. Thus, more research is needed on enrolling postpartum women into clinical trials and supporting them postdelivery to prevent relapse to smoking.

Currently, few treatments have shown efficacy at reducing postpartum relapse to smoking. Treatments include behavioral approaches, (e.g., Cognitive Behavioral Therapy and self-help mailings) [11–14] and nicotine replacement therapy [15, 16]. Women's use of these treatments for postpartum relapse prevention is limited. Given the efficacy of pharmacotherapy for relapse prevention outside of pregnancy, more work is needed to expand relapse prevention pharmacotherapy options for those who are postpartum [3, 17–19]. Non-nicotine-containing medications may be indicated for postpartum women who have been abstinent from nicotine during pregnancy due to reluctance to reintroduce nicotine. Bupropion is worth consideration for use in postpartum relapse prevention because it has demonstrated efficacy as a smoking cessation aid [20] and is generally safe and well-tolerated, with few side effects [21]. Bupropion can further reduce nicotine cravings and improve depression, both of which are associated with increased risk for smoking relapse [22]. For pregnant women, postpartum depression is of particular concern, with up to 20% developing the disorder [20, 23, 24]. Thus, bupropion may be particularly effective at preventing postpartum relapse, in part, because of its antidepressant effects.

One potential concern for use of bupropion for postpartum relapse is the safety for infants who are breastfeeding. LactMed (last updated July 2022), in summarizing data regarding the use of bupropion during lactation, reported that maternal bupropion use results in low concentrations of bupropion and bupropion metabolites in breast milk which would not be expected to cause adverse events in breastfed infants [25]. Bupropion has been studied for postpartum depression in a small sample of women, some of whom were breastfeeding. Bupropion use was not associated with adverse effects on infant health or lactation [24]. Investigational use of the medication was approved by the U.S. Food and Drug Administration as part of the Investigational New Drug application for this study.

Although there is a need to investigate treatments for prevention of postpartum relapse, clinical trial recruitment for postpartum relapse prevention studies can be quite difficult [26]. The most successful recruitment methods for pregnant individuals in the literature include multiple streams of engaging participants, including using clinical registries, mailings through healthcare settings, traditional media, and more recently social media [26]. Despite these methods, few clinical trials targeting pregnant and postpartum women meet their recruitment target in the time originally planned [27]. Previous research has reviewed struggles with recruitment [26, 28–30]. Medication trials in pregnant and postpartum women, especially those choosing to breastfeed, may have unique recruitment challenges including greater reticence to accept pharmacological intervention than the general population [31]. A trial utilizing nicotine replacement therapy among pregnant women identified five main recruitment challenges: low smoking disclosure rate, smoking too few cigarettes, late entry into prenatal care, small potential participant pool, and a high refusal rate [29].

In addition to recruitment being difficult, it is also expensive. For instance, one study investigated the costs of recruiting individuals to join research in which participants received relapse prevention self-help materials and self-report questionnaires. Various strategies such as media advertisements, direct mailings, and healthcare provider outreach were high cost with low yield. Media advertisements resulted in a cost of $6,332 per participant. Outreach calls were successful, but costly as paid staff worked approximately 200-250 hours per month for 1.5 years to successfully recruit 338 people [28].

While previous studies have enumerated reasons for low participant accrual and suggested methods for recruitment, very few qualitative studies have been conducted to determine how pregnant women view participation in smoking cessation and relapse prevention research during the postpartum period. Qualitative interviews may be particularly helpful for identifying potential barriers to recruitment and understanding the perceptions of potential participants compared to survey methods or record review, which rely on investigator generated hypothesized barriers [32].

We are currently conducting a clinical trial testing bupropion for postpartum relapse prevention (BurPPP) [17]. Like previous research involving the recruitment of pregnant women, participant identification and engagement has been challenging. Over the initial two years of the study, we have been contacted by or reached out to 845 women to assess interest/eligibility. Of these, 135/835 (16%) were screened, 76/835 (9%) were eligible, and only 16/835 (2%) enrolled. Given this low rate of accrual, we conducted exploratory qualitative interviews to assess pregnant women's interest in (1) remaining abstinent postpartum, (2) using bupropion for relapse prevention, and (3) participating in research studies to address smoking relapse following prenatal abstinence. In this qualitative investigation, we sought to gain insight into pregnant individuals' attitudes regarding research participation to inform recruitment strategies for future postpartum relapse prevention studies.

## 2. Methods

### 2.1. Human Subjects

This study was conducted with approval via the University of Minnesota's Institutional Review Board (IRB; #00007684, approved November 1, 2019).

### 2.2. Recruitment

Potential participants were contacted as a part of a larger clinical trial testing the efficacy of bupropion for prevention of postpartum relapse [17]. Participants were recruited from April 2021 to November 2021. Participants were identified and recruited via the following methods for the full trial and were then asked if they were interested in participating in the qualitative study: (1) letters sent to pregnant individuals in outpatient and inpatient with a smoking history seen at OB/GYN or Family Medicine clinics, (2) self-referral via Facebook ads for the BurPPP trial, (3) advertising across social media platforms managed by a clinical research recruiting firm [33], and (4) in-person recruitment medical settings. Interested individuals were screened for eligibility over the phone. For participants who were eligible for the qualitative study, they were read and sent a study fact sheet conforming to the elements of informed consent and verbal consent was obtained prior to conducting the interviews.

### 2.3. Participants

Eligible participants were pregnant women who were between 18 and 41 years of age, English speaking, and who endorsed that they either (1) quit smoking during their pregnancy or (2) were considering quitting smoking during their pregnancy. Of note, participant advertisements detailed participation in the larger trial, and they were informed of the qualitative interview during the initial contact. They did not have to be interested in participating in the larger trial to complete the interview.

### 2.4. Qualitative Data Collection

After obtaining verbal consent, a Bachelor's-level tobacco treatment specialist (MAH) completed a semistructured interview. The interview guide (Appendix A) gathered general demographic information and assessed attitudes, knowledge, and beliefs surrounding the following topics: (1) pregnancy and quitting, (2) postpartum cessation, and (3) participation in research generally and the BurPPP study, specifically bupropion vs. placebo for postpartum relapse prevention.

All interviews were conducted via telephone and were audio recorded. On average, interviews lasted between 15 and 25 minutes. Participants were compensated with a $25 gift card for their time.

### 2.5. Data Analysis

Using NViVo Pro, two independent raters (MAH and PF) double-coded all interview transcripts using thematic analysis. A codebook was created and codes refined during meetings between raters. Themes were reviewed and considered in context of previous work. A final codebook was created upon agreement by both raters.

## 3. Results

### 3.1. Demographics

All participants (*N* = 22) identified as female and were between the ages of 22 and 41 (73% White). Most had completed high school/GED. Monthly household income ranged from $0 to $8,000 with a median monthly income of $1,766 and average of $2,967. For 36% of women, it was their first pregnancy. At the time of the interview, 59% of the participants had quit smoking. See Table 1 for demographics.

### 3.2. Perceptions regarding Smoking during Pregnancy

Overall, women were extremely knowledgeable about the risks of smoking during pregnancy and motivated to quit/stay quit. They typically received information on these risks from friends, family members, social media, traditional media, and clinicians. Most participants were certain that quitting smoking was the best decision for their unborn child.

Despite the overall interest in quitting, several participants expressed skepticism about the importance of quitting. These participants told anecdotes about their own previous pregnancies or the pregnancies of other women in which the children had no postpartum difficulties even if the mother had smoked throughout pregnancy.

*“I'm in a Facebook group with a lot of smoking soon to be moms and I mean, everybody has been pretty healthy and being born pretty healthy and stuff like that.”* (24-year-old White woman)

The perception of being judged for smoking while pregnant varied by smoking status. For those who quit smoking at the beginning of their pregnancy, none reported feeling judged for being a former smoker and many reported experiencing praise for successfully quitting. For those who had difficulty quitting, some reported experiencing judgement, particularly from partners. A couple of women noted that they themselves would judge others if they had smoked during their pregnancy and were unable to quickly quit.

*“I haven't felt personally judged. But if I knew other people that were smoking in pregnancy, I would judge them.”* (28-year-old White woman)

### 3.3. Smoking Cessation during Pregnancy

Participants varied in smoking status at the time of the interview and timing of cessation during pregnancy. Some women quit as soon as they found out they were pregnant (45%), while others quit later in pregnancy (14%), and others still were unable to successfully quit (41%). The majority of abstinent women indicated they quit cold turkey. Many mentioned strategies for staying quit, such as delaying cigarettes or distracting themselves during a craving. For those that had tried nicotine replacement therapy, use was often short lived (e.g., casual use and putting the patch on a few times over a week). This is in line with previous research which has found overall reticence and underuse of nicotine replacement therapy in pregnant women [34, 35]. One person noted their provider talked to them about quitting but had not assisted in making a plan or prescribing medication to help fully quit.

### 3.4. Barriers and Facilitators to Quitting Smoking during Pregnancy

All women expressed some interest in quitting; however, many indicated barriers to quitting successfully. Barriers included personal life stressors as well as partners, friends, and family who smoked. For participants with other children, some described smoking as one of the few times of day when they were alone. For those who had not been able to quit during their pregnancy, most had reduced their cigarettes per day.

Overall motivations for quitting during pregnancy were twofold: (1) personal reasons outside of pregnancy for quitting and (2) concern for the baby's health. Unrelated to their pregnancy, many women told us that they had already attempted to quit or were starting to think about quitting ahead of finding out they were pregnant. Motivations for quitting smoking prior to pregnancy included money, time, and appearance. The women who were able to quit at the beginning of pregnancy noted that the smell of cigarettes was no longer appealing while pregnant and even exacerbated morning sickness. They noted their aversion to the smell of cigarettes made it easier to quit. For many, there was also strong desire to be as healthy as possible for their baby.

*“… It was really exciting news to find that [pregnancy] out and [I] didn't want to do anything to compromise that.”* (31-year-old White woman)

### 3.5. Postpartum Smoking Plans

Most women, though with some trepidation, planned on staying smoke-free postpartum. They viewed pregnancy as a means to quit for good. Some women, particularly first-time mothers, noted that lifestyle changes associated with parenthood (e.g., not going to bars or clubs where it is tempting to smoke) would make it easier to stay quit.

Overall, participants were quite confident that they would be able to quit and stay quit without assistance. This was true even for participants who had not remained abstinent after previous pregnancies and for those who had not yet quit. These women indicated that staying quit would be difficult, but they believed that this time would be different.

*“I don't plan to smoke after I give birth. And I'm just hoping I can stick it out and not you know, like, crave it anymore.”* (23-year-old White woman)

### 3.6. Attitudes towards Research

Participants overwhelmingly had positive feelings towards research. A few individuals had participated in studies or had some knowledge of research methods from their education. Many expressed enthusiasm for the prospect of helping other pregnant women through research.

*“I really liked the idea… It's helpful for people. I know that a lot of my friends and family… when they're pregnant, they don't care if they smoke. You know, because they probably don't know the information about it.”* (37-year-old Native American woman)

By far the biggest reason for disinterest in participation in our trial was concern about taking bupropion. A number of women had heard of bupropion and were aware of its uses for mood as well as some of its side effects. Those who knew little about bupropion were quite concerned about side effects for themselves as well as risk to nursing infants. Two individuals noted they would prefer to receive the placebo pill as they were worried about taking medication. Even participants who were generally open to taking medication noted that taking medication for a study was unappealing.

*“But like maybe people are just scared. You know, like, the medication might have like, some sort of side effects that they didn't know about or something like that.”* (24-year-old White woman)

Overall, women who were planning on breastfeeding were the most concerned about taking the medication. Though as part of the Investigational New Drug (IND) process, our medical team, the IRB, and the U.S. Food and Drug Administration (FDA) determined the amount of bupropion transferred to the baby via breast milk was unlikely to cause harm, participants were still concerned about risk to the baby [36, 37]. Many expressed hesitation about taking bupropion or a desire to discuss the medication with their healthcare provider.

*“I don't want that medication in the breast milk either. I just would, would rather not. And that's my own… I know that they say it's safe, I just, I'm so hesitant on it.”* (34-year-old White woman)

Outside of the specific medication for the study and concerns about taking medication while nursing, a number of participants expressed hesitancy about medication use generally and skepticism about chemicals in pills. None of these individuals drew a comparison between the chemicals in medications to chemicals in cigarettes. Many expressed particular disinterest in taking medication for relapse prevention because they did not believe they were at risk for relapse.

*“I'm not too fond of taking meds, trying pills and stuff. I don't know, I get this anxiety when it comes to taking medication.”* (41-year-old Black woman)

Similarly, the second most common reason for lack of interest in the study was that for those who had quit or were confident they would quit during their pregnancy they found little reason to be in a relapse prevention study.

*“I mean, I hate to say it, but like, I would be a very bad test subject, because like, I have no interest in picking up another cigarette like... The want is already gone.”* (33-year-old White woman)

We asked participants about ways to structure the study to make them more likely to participate. Overall, participants were concerned about making new time commitments while beginning parenthood. Due to time concerns, there was a preference for online surveys over visits and virtual over in-person visits. Some expressed interest in a person to talk to for accountability purposes.

*“I just didn't know if I could commit to those weekly check-ins and you know, having the time to sit down and do a 60-minute interview via zoom, you know, once a week like that.”* (31-year-old White woman)

Compensation was not a large motivator for study participation. However, participants preferred larger compensation increments over fewer visits or a shorter timeframe.

## 4. Discussion

In this qualitative study of perceptions of participation in postpartum relapse prevention research, we found that interest in quitting smoking and staying quit is high among women in pregnancy and postpartum. There is some interest in behavioral support; however, these interests were overshadowed by the burden of time associated with said support. Attitudes towards research in general were positive; however, interest in participating in relapse prevention studies was low, partly due to the burdens of new motherhood.

Women in our study overestimated their likelihood of staying quit postpartum, with nearly all believing they would be able to remain abstinent. In epidemiological studies, approximately half of women quit smoking during pregnancy [4, 38]. However, in the first year postpartum, most women will relapse (within the first six months 40-50% may relapse, [6, 7] while at twelve months relapse rates climb to 70-90% [4, 8, 9, 39, 40]). Women reported getting information about the importance of abstinence during pregnancy from family, friends, media sources, and their healthcare team. Thus, healthcare providers appear to be a trusted information source for communicating risk of relapse as well as the risks and benefits of pharmacological and behavioral interventions. Similar research has also shown healthcare clinicians as trusted resources for relapse prevention [41, 42]. Educating pregnant individuals on their high risk of relapse may increase interest in postpartum relapse prevention treatments.

In general, pregnant women were reluctant to take medication to stop smoking and/or stay quit postpartum. This attitude was particularly true for women who planned to breastfeed. Prior literature is consistent with these findings, showing that postpartum individuals are unlikely to use pharmacological treatments for tobacco dependence [31, 42, 43]. The Health Belief Model may partially explain this. The Health Belief Model states that health behavior is guided by the motivation to avoid illness (smoking-related disease or adverse effects on child health) and the belief that engaging in a particular behavior will prevent illness. According to the model, the likelihood of engaging in a health behavior (e.g., taking medication to prevent relapse) is a combination of the perceived severity of the illness, the perceived susceptibility to illness, the perceived benefit of engaging in the behavior, and the perceived barriers of engaging in the behavior. In this case, pregnant women did not feel they were susceptible to illness because they did not believe they would relapse, and they perceived the barriers of taking medication (potential adverse effects to mother and child) to be too great [44, 45]. Patients may benefit from decision aids from providers and community members, step-up treatments if they do relapse, and detailed education regarding the risk and benefit of the pharmacologic interventions.

Cessation medication hesitancy is concerning due to research showing that use of smoking cessation medication doubles quit rates [46]. While small amounts of nicotine from nicotine replacement therapies is transferred to infants during breastfeeding, the amount of nicotine transferred is substantially less than if the parent continued to smoke [36, 37, 47, 48]. In the same vein, bupropion doses are transferred in small amounts to infants via breast milk (reported to be 2% of weight-adjusted maternal dose of bupropion and its active metabolites) [49], and the benefit of abstinence from cigarettes vastly outweighs the small risks of taking the medication [50]. Some of the risks of smoking while breastfeeding include disturbed sleep and potential for damage to the liver and lungs [51, 52]. Additionally, secondhand smoke is associated with asthma, chronic respiratory infections, SIDS, and behavior modeling, as well as a number of other adverse effects on the child [4, 10, 53]. It is important to note that the recommendation by LactMed for bupropion in breastfeeding women is “use with caution” [50]. Women need help weighing the known harms of infant smoke exposure versus the harms of short-term medication use [50]. It is possible that relapse prevention treatment would be more acceptable to postpartum women if offered after they begin to struggle maintaining abstinence rather than if offered prophylactically.

While women were supportive of research, few women were interested in signing up for our postpartum relapse prevention study. This finding is consistent with the literature on recruiting pregnant smokers [26, 28, 29]. To our knowledge, our study is the first to ask women why they are uninterested in participating in relapse prevention research using medication. Women noted high confidence in remaining quit and low interest in taking medication and concerns about study burden as reasons not to participate. Many were specifically concerned with taking on new commitments postpartum. Participants suggested reducing participant burden with online participation, reduced visit numbers and length, and better compensation. However, even low contact, low burden, studies of postpartum relapse prevention treatments have had difficulty recruiting [28]. Thus, it could be that misperceptions about relapse risk coupled with reluctance to make commitments postpartum may be combining to drive poor recruitment success.

This study should be interpreted with limitations in mind. This is a small qualitative study of women in a single state who were mostly White. Their expressed opinions may not be representative of the experience of individuals in other geographic regions or from different racial/ethnic backgrounds. This study was designed primarily to gain insight into the poor recruitment success of the BurPPP trial. The interview guide was developed by the researchers and not informed by a guiding theory or community input, which would have strengthened the scientific rigor of the study. However, our experience is consistent with other studies that have shown difficulty recruiting pregnant and postpartum women into relapse prevention studies [26, 28, 29]. Future work should investigate partner perspectives, which are an important factor in postpartum relapse [54].

This study serves to add insight to this discourse. It is worth noting that our trial recruitment may have been particularly difficult due to the COVID-19 pandemic. However, in interviews, most women reported that COVID-19 would not affect their interest in a remotely conducted clinical trial. Finally, our study only asked about interest in bupropion and not other medications for cessation. It may be that women are more willing to take other medications, such as nicotine replacement therapy [26]. However, many women in interviews noted a reticence to take medication generally, not just bupropion.

In conclusion, postpartum relapse to cigarette smoking poses significant health risk to women and children but few women believe they will relapse. Barriers to engagement in relapse prevention trials include reluctance to take medication and to take on additional time commitments postpartum. Healthcare systems may be in a position to offer reimbursement for childcare costs or additional parental supports to address concerns about the maternal burden that might come from engaging in smoking cessation activities. Healthcare clinicians are trusted advisors and may be one conduit for promoting engagement in cessation programs by educating about relapse risk vs. medication risk or by helping to catch early relapse behavior and connect patients to treatment. While specific solutions to these issues are beyond the scope of this study, addressing the concerns that these women expressed may be critical to optimizing participation in effective relapse prevention research and programs.

## Figures and Tables

**Table 1:** Participant characteristics (*N* = 22).

	*n* (*M*)	% (SD)
*Female*	22	100%
*Education*		
Some high school	3	14%
High school/GED	9	41%
Some college	2	9%
College	8	36%
*Age*	(30.09)	(5.86)
*Race*		
American Indian/Alaska Native	1	5%
Black/African American	2	9%
Caucasian/White	16	73%
Hispanic/Latino	1	5%
Other	2	9%
*Income ($/month)*	(2,967)	(2,546)
*First pregnancy*		
Yes	8	36%
No	13	59%
Missing	1	5%
*Quit status (at time of interview)*		
Yes	13	59%

## Data Availability

Due to the nature of this research, participants of this study did not agree for their data to be shared publicly, so supporting data is not available.

## Supplementary Materials

Supplementary Materials Appendix A: interview guide. This list of questions (created by MAH and JT) guided the conversation with participants during qualitative interviews.
