# Supplementary material for: Knowledge, Attitudes, and Beliefs about Relapse Prevention Research Involving Bupropion among Current and Former Pregnant Individuals Who Smoke
Source: J Smok Cessat. 2022 Dec 16;2022:1925071. doi: 10.1155/2022/1925071 (PMC9788884; doi:10.1155/2022/1925071)
Supplement: Supplementary Materials — Appendix A: interview guide. This list of questions (created by MAH and JT) guided the conversation with participants during qualitative interviews. [file 1925071.f1.docx]

**Appendix A: Interview Guide**

| **Theme** | **Question** | **Follow-up question** | **Probe** |
| --- | --- | --- | --- |
| ***Pregnancy & quitting*** | 1. How has this pregnancy been for you? | a. Is this your first pregnancy? | |
|  |  | b. How are you feeling about being a new parent? | |
|  |  | c. Are you planning on nursing? | |
|  |  | d. Any concerns at this time? | |
|  | 2. What do you know about smoking in pregnancy? | a. Have you felt any judgement about your smoking and pregnancy? | |
|  | 3. I know you’re thinking of quitting during your pregnancy. Have you already quit smoking? | | |
|  | 4. (If yes to question 3) Tell me about your choice to quit smoking during pregnancy. | a. When did you quit smoking for this pregnancy? | What week or trimester, or did you quit when you were trying to get pregnant? |
|  |  | b. Did you find it difficult to make the decision to quit? | What made you decide to quit? What are your beliefs about smoking and children’s health? |
|  |  | c. How did you quit? | Sometimes, people might use things like the nicotine patch, lozenge, or medication, and sometimes people quit cold turkey or by using other methods. |
|  | 5. (If no to question 3) How is your quit attempt going for you during this pregnancy? | a. Did you find it difficult to make the decision to quit? | |
|  |  | b. What made you decide to quit? | What are your beliefs about smoking and children’s health? |
|  |  | c. Is there anything you are currently using or planning on using to quit? | Sometimes, people might use things like the nicotine patch, lozenges, or medication, and sometimes people quit cold turkey or by using other methods. |
| ***Postpartum cessation*** | 6. What are your plans for smoking after your baby is born? Do you plan to stay quit? Why or why not? | a. How difficult do you think it will be to stay smoke free after your baby is born? | |
|  |  | b. What do you think might make staying quit difficult? | Examples – Do you live with smokers, stress, anxiety, feeling depressed, weight concerns |
|  |  | c. What do you think might make staying quit easier? | Examples – Social support, motivation, new baby; Would you be interested in support, if so, what kind? |
|  |  | d. Would you be interested in a medication after your baby is born that could potentially help you stay quit? | |
|  | 7. (If no to 1a) I want to talk a bit about your other pregnancies. Did you quit during those? | | |
|  | 8. (If yes to 7) What was that like for you? |  | Was it similar to the quit during this pregnancy? Was it easier/harder? If so, why do you think that was? Did you use a medication to help you quit that time? |
|  |  | a. How long after your baby was born did you stay quit? | |
|  |  | b. What do you think brought you back to smoking? | Sometimes people say things like stress, anxiety, social circle, etc. |
|  | 9. (If no to 7) Did you plan to quit during your other pregnancies? | a. (If yes to 9) What made quitting tough during your other pregnancies? | |
| ***Research feelings and BurPPP project opinions*** | 10. In general, what are your feelings about participating in a research study? |  | Research might include things like taking a new medicine, taking a survey, etc.; If you have participated in the past, tell me about your experience. |
|  | 11. *Explain study to participant-* We are asking pregnant individuals who have quit smoking during their pregnancy to either take a medication or a sugar pill, called a placebo, for 12 weeks after they give birth. We hope this medication can help pregnant individuals stay smoke free after their baby is born. |  |  |
|  | 12. We’ve had trouble recruiting people to participate and we don’t fully understand why. How does that study sound to you? |  | What do you not like about the study? |
|  | 13. What would you hold you back from participating in something like that? |  | I know when we talked earlier, you said *XYZ* |
|  | 14. What concerns do you have about taking a medication after delivery? | a. Does your decision about nursing make you more or less likely to want to participate in a project about medication use postpartum? | |
|  | 15. Like I mentioned, some individuals will get a medication and some will get a sugar pill. So there is a 50/50 chance, similar to flipping a coin, that a person will not get a medication. Would the chance that you may not get the medication affect your interest in participating in the study? |  |  |
|  | 16. If you were able to get some kind of additional support after giving birth, like counseling, how would that change your willingness to participate? |  |  |
|  | 17. All of our procedures and study visits are done remotely over the phone, telemedicine type visits, online and by mail. What do you think about this? Would you prefer virtual visits or in-person visits? Why? |  | Examples – Technology challenges, home environment |
|  | 18. We are checking in with people once during their pregnancy and then weekly for the first 12 weeks after giving birth, would that work for you? |  |  |
|  | 19. We would check in again at months 6, 9, and 12 after birth. What do you think of this schedule? |  | Is this too much or not enough? |
|  | 20. Right now, we are offering women $860 to participate. What are your thoughts on that? |  |  |
|  | 21.What do you think would make you more likely to participate in a project like ours after giving birth? |  | Examples – Payment, child care, in-person visits/paid transportation |
|  | 22.Does the current coronavirus pandemic impact your interest in doing a research project right now? Why or why not? |  |  |
| ***Other*** | 23. Is there anything else we didn’t cover that you’d like to tell me? |  |  |
